# Impact of polymorphic transposable elements on transcription in lymphoblastoid cell lines from public data

**DOI:** 10.1186/s12859-019-3113-x

**Published:** 2019-11-22

**Authors:** Giovanni Spirito, Damiano Mangoni, Remo Sanges, Stefano Gustincich

**Affiliations:** 1 Area of Neuroscience, Scuola Internazionale Superiore di Studi Avanzati (SISSA), Trieste, Italy; 2 Central RNA Laboratory, Istituto Italiano di Tecnologia (IIT), Genoa, Italy; 3 Biology and Evolution of Marine Organisms, Stazione Zoologica Anton Dohrn, Naples, Italy

**Keywords:** Quantitative trait loci, Transposable elements, Functional enrichment

## Abstract

**Background:**

Transposable elements (TEs) are DNA sequences able to mobilize themselves and to increase their copy-number in the host genome. In the past, they have been considered mainly *selfish DNA* without evident functions. Nevertheless, currently they are believed to have been extensively involved in the evolution of primate genomes, especially from a regulatory perspective. Due to their recent activity they are also one of the primary sources of structural variants (SVs) in the human genome. By taking advantage of sequencing technologies and bioinformatics tools, recent surveys uncovered specific TE structural variants (TEVs) that gave rise to polymorphisms in human populations. When combined with RNA-seq data this information provides the opportunity to study the potential impact of TEs on gene expression in human.

**Results:**

In this work, we assessed the effects of the presence of specific TEs in *cis* on the expression of flanking genes by producing associations between polymorphic TEs and flanking gene expression levels in human lymphoblastoid cell lines. By using public data from the 1000 Genome Project and the Geuvadis consortium, we exploited an expression quantitative trait loci (eQTL) approach integrated with additional bioinformatics data mining analyses. We uncovered human loci enriched for common, less common and rare TEVs and identified 323 significant TEV-*cis*-eQTL associations. SINE-R/VNTR/Alus (SVAs) resulted the TE class with the strongest effects on gene expression. We also unveiled differential functional enrichments on genes associated to TEVs, genes associated to TEV-*cis*-eQTLs and genes associated to the genomic regions mostly enriched in TEV-*cis*-eQTLs highlighting, at multiple levels, the impact of TEVs on the host genome. Finally, we also identified polymorphic TEs putatively embedded in transcriptional units, proposing a novel mechanism in which TEVs may mediate individual-specific traits.

**Conclusion:**

We contributed to unveiling the effect of polymorphic TEs on transcription in lymphoblastoid cell lines.

## Background

Transposable elements (TEs) are genomic sequences able to move from one location to another within the genome [1]. TE activity throughout eukaryotes evolution increased the number of their copies in the genome to the point where approximately 45% of the human genome is constituted by TE-derived sequences [2]. Their contribution is likely to be even larger since the identification of ancestral TE-derived sequences is made harder by the accumulation of mutations over time. Despite being widely considered *junk DNA* at first, increasing evidence is showing that TEs are involved in major developmental and evolutionary functions [3, 4]. In particular, TEs have been often shown to contribute to the regulation of flanking or overlapping genes by supplying *cis*-regulatory sequences [5–9]. Namely, in human, TE-derived sequences provide functional elements to promoters [6, 10, 11], enhancers [12–14], transcription terminators [15] and splice-sites [16, 17]. Moreover, a single human TE can contribute to the regulation of multiple genes by influencing chromatin structure [10]. Furthermore, TE embedded in transcripts can have a role in the regulation of translation [18] and RNA editing [19]. Indeed, a substantial portion of 3′-UTR-located Alu elements is believed to be at the basis of regulatory modules that, throughout primate evolution, stabilized their presence in the genome as miRNA binding sites [18]. Alu elements may also drive the diversification of the proteome by inducing A-to-I site-selective RNA editing [19], a process widespread in primate transcriptomes [19, 20]. Finally, specific TEs are also essential for post-transcriptional regulation of some coding genes trough antisense lncRNAs [21, 22].

It is therefore likely that throughout human evolution different TE-derived sequences have been co-opted into diverse classes of functional elements [12, 23]. Interestingly TE-derived regulatory sequences are enriched nearby genes involved in immunity, response to external stimuli, neurodevelopmental functions [12] and overall lineage- or species-specific genes [6]. This is consistent with the fact that single TEs are mostly lineage-specific genomic features, and are therefore prone to impact fast-evolving species-specific phenotypic features. Conversely, TEs are depleted nearby conserved genes [12]. Taken together, these observations suggest that TEs may have contributed substantially to the evolutionary diversification of mammalian genomes presumably by generating lineage-specific patterns of gene regulation [6, 24]. In this regard, autonomous and non-autonomous human young and currently active TEs result of particular interest.

The only classes of TEs believed to be still active in the human genome are the long interspersed nuclear elements (LINE) L1, the short interspersed nuclear elements (SINE) Alu and the SINE-VNTR-Alu (SVA) [2]. These elements are transcribed and mobilize through a copy-and-paste mechanism called retrotransposition, increasing the number of their copies in the genome. Full length L1 elements are about 6 Kbp long, they make up about 17% of the human genome [25] and are the only known autonomous TEs in human [2]. In addition to having an impact on the genome through their own retrotransposition, L1 s protein machinery can cause the mobilization of non-autonomous transcribed TEs such as Alu and SVA [26] and may also cause the birth of pseudogenes [27]. Alus elements are only a few hundreds nucleotides long [28], they are unique to primates and, being the most common class of SINE in the human genome, they represent a major factor of genetic diversity [29–31]. Similarly, SINE-VNTR-Alu (SVA) elements are the evolutionarily youngest class of human TEs. Due to their multi-domain composition, these elements have been described as *composite non-coding retrotransposons* [32]. Each domain is believed to derive from either a retrotransposon or a simple repeat sequence [32]. Canonically, starting from the 5′ end an SVA element is composed by: an hexameric CCCTCT repeat, followed by sequence sharing homology to two antisense Alu fragments, a variable number of GC-rich tandem repeats (VNTR), a sequence likely derived partially from the ENV gene and partially from the 3′ end of a repeated sequence of an ancient endogenous retrovirus (HERV-K10) [33], and a polyadenylation signal. They have been found to lay more densely in gene-rich regions rather than gene deserts [32], to be able to alter gene expression of flanking genes [32, 34, 35] and cause diseases [36]. The young evolutionarily age of SVAs combined with their ability to alter gene expression could lead to the question whether these mobile elements played a role in the evolution of the central nervous system (CNS) and the emergence of functional trends characteristic of the human species. Indeed, functional enrichment analyses of genes associated with SVA elements in the human genome showed an association to Gene Ontology functional categories related to brain functions and disorders [35]. Due to their activity in recent and current times, these three families of TEs generated variation among human genomes. When a new copy of these elements results harmless or even provide advantages for the host genome, it may segregate as common variant and increase its frequency in a population, becoming therefore a polymorphism [37]. Population surveys analyses have indeed uncovered thousands of individual TE insertions which putatively evaded purifying selection and were shared among multiple individuals [38, 39], often belonging to the same population [40]. In this work, we will refer to these genomic variants as Transposable Elements Variants (TEVs).

The identification of TEVs and their effects on gene expression requires the sequencing of several individual genomes and transcriptomes, which only the most recent sequencing technologies have allowed. In particular, the 1000 Genome Project, upon sequencing full-length genomes of more than 2000 individuals from 25 worldwide populations, stored and released all recorded data and results on public databases [38]. The main aim of this consortium was to provide a comprehensive catalog of multiple classes of human genomic variants [38]. Indeed, the final release of the project (phase III) described over 88 million variants (84.7 million SNPs, 3.6 indels and over 60,000 structural variants) [38]. Despite being the lowest in number, structural variants (SVs) represent the majority of varying nucleotides among human genomes. SVs uncovered by 1000 Genome Project phase III include about 18,000 TEVs [38]. Alongside the 1000 Genome Project, the Geuvadis consortium performed both RNA and small RNA sequencing on lymphoblastoid cell lines (LCL) of 445 individuals who participated to the 1000 Genome Project [38, 41]. It is therefore possible to integrate these individual genomic and transcriptomic datasets in order to assess the impact of genomic variants on gene expression in this cell line. These kinds of analyses are performed by regressing gene expression levels for individual samples against locus-specific genotypes for matched samples [42, 43], and they are usually referred to as expression Quantitative Trait Loci (eQTL). Several studies so far associated specific loci to the variation in RNA expression levels in human [44] and other species [45–47]. One of the most comprehensive eQTLs study has been performed by integrating the 1000 Genome Project data with the Geuvadis dataset [48]. Here the majority of analyzed genes showed substantial variation in expression levels linked to genomic differences among individuals [48]. Even though the eQTL method was originally developed for SNPs data [49], the 1000 Genome Project dataset also allowed to perform eQTL analysis related to SVs. Interestingly, these studies revealed a disproportionate impact of SVs on gene expression relative to their number (compared to other classes of variants), meaning that they have a higher probability of being the causative variant in an association [41]. One of the most common type of SVs in the human genome are TEVs. Due to the established role of TE-derived sequences as regulatory modules in the human genome, it has been proposed that TEVs could give rise to individual- and population- specific gene regulatory innovations. A recent work presented a population-level view of the regulatory consequences of TEVs through an eQTL approach revealing several associations between the presence of common TEVs (> 5% allelic frequency) and altered gene expression levels [40]. While the results of this and other eQTL analysis were cell-type specific, recently completed projects, such as the GTEx project [50], has provided SNP-based eQTL data from multiple tissues of hundreds of individuals uncovering the extent and the potential effects of genetic variation on human transcriptomes of different organs [44]. These studies have focused their attention on TEVs with minor allele frequency (MAF) > 5%, while excluding less common and rare variants.

In this work, we associated polymorphic TEs to changes in gene expression levels of flanking genes using a *cis*-expression quantitative trait loci (*cis*-eQTL) approach [49] taking advantage of the data generated in the 1000 Genome [38] and the Geuvadis [48] project. Our works extends previous similar analysis [40] as our dataset includes TEVs with MAF < 5% in the analyzed population and also identify possible transcriptional structural polymorphisms mediated by TEs. We present an initial assessment of the impact of less common and rare TEVs on gene expression since variants with these frequencies have been proposed as potential source of missing heritability and responsible for a portion of the uncharacterized genetic risk underlying many diseases. Genomic regions strongly enriched in TEVs and associated with expression level alteration of flanking genes are herein described. Furthermore, we pointed out differences in localization, impact and functional relevance of the three classes of TEs active in human (Alu, L1, SVA) and explored the possibility that some of them might be transcribed as part of known transcripts.

## Methods

### Data used

Coordinates and genotype information about polymorphic TEs regarding 445 healthy individuals were taken from the phase 3 variant release of the 1000 Genomes Project [38]. Coordinates referred to the human genome reference sequence build GRCh38/hg38. Genotype calls were performed on a Epstein–Barr virus (EBV) transformed B-lymphocytes cell line (LCL) as described in the 1000 Genome Project structural variation main publication [38, 39]. The TEV genotype data were accessed from the 1000 Genomes Project ftp at the NCBI: ftp://ftp.1000genomes.ebi.ac.uk/vol1/ftp/phase3/integrated_sv_map/.

RNA-seq gene expression data from LCL of the same 445 individuals, was taken from the Geuvadis RNA sequencing project [48]. All RNA-related data were obtained as described in the Geuvadis main publication [48]. RPKM gene expression data used in this work was downloaded from the Geuvadis project ftp server at EBI: ftp://ftp.ebi.ac.uk/pub/databases/microarray/data/experiment/GEUV/E-GEUV-1/analysis_results/.

SNP-based eQTL data from 44 human tissues from 449 individuals has been downloaded from the GTEx project (V7) main website: https://gtexportal.org/home/datasets. In order to assess which genomic segments are most dense in eQTLs, we intersected coordinates of eQTLs (hg19) with coordinates of human karyotype bands (hg19) from UCSC (http://hgdownload.soe.ucsc.edu/goldenPath/hg19/database/cytoBand.txt.gz) with bedtools [51]. We then normalized the eQTL count for the size of each karyotype band. This experiment was performed both for all 44 tissues and for 14 sets resulting from the grouping of the 44 tissues. Grouping was performed as shown in Additional file 1.

### Sliding windows enrichment analysis

TEVs are spread throughout the genome. We wanted to assess whether some genomic regions are enriched in TEVs in comparison to a random model. We therefore searched for enrichments by applying a sliding window approach. We divided the human genome (release hg38) into sliding windows of 10Mbp overlapping by 5Mbp. The sliding windows method is a fairly common approach when examining large genomic data [52] as it presents some advantages when analysing regions that are arbitrarily chosen. Upon generating a bed file containing the genomic coordinates of the windows with a custom python script, hg38 TEVs coordinates were intersected with it using bedtools [51] separately for each TE class; subsequently the number of overlaps for each window has been plotted. To assess whether the number of TEVs in each window was significantly higher compared to the rest of the genome, the amount of TEVs in each window was compared with the result of the same experiment performed with a set of random coordinates of the same number and length of the TEV classes. Then Z scores were calculated for each window, and windows featuring a Z score above 3 were considered to be enriched for that class of TEV. The same analysis has also been performed for TEV putatively involved in gene expression regulation through eQTLs.

### *cis*-eQTL identification

In order to assess the impact of TEVs on gene expression we associated previously described genotype and RNA-seq data of 445 individuals from the 1000 Genome Project [39] and the Geuvadis consortium [48] to produce *cis*-eQTLs using a custom python script. Of the total 11,579 TEVs resulting from the analysis of 445 healthy individuals, we selected only those present in at least 3 individuals resulting in 8551 used for this analysis. Each cis-eQTL represented an association between a TE polymorphism and a gene in a range within 1 Mbp which showed a significant alteration in its expression levels in individuals carrying the polymorphisms compared to individuals not carrying it. These alterations have been identified using the program Matrix eQTL [53] with the additive linear (least squares model) option and covariates for gender and population for all analysed individuals, keeping also genotypes into account (0, 1, 2). While this operation was performed for all possible pairs of TEVs and genes, we took into account only association in *cis* featuring a distance between the gene and TEV below 1 Mbp. Finally, we selected as significant *cis*-eQTL only those showing an FDR < = 0.05 which resulted in the identification of 323 associations. The significance of TEV-*cis*-eQTLs was also assessed through randomization: for a total of 100 randomizations, each TEV genotype has been randomized. Real data showed 323 significant results (FDR < = 0.05), which is significantly higher than the average number of significant results from the 100 randomizations (mean number of significant results = 76.5, Z > 50). We also compared the TE-*cis*-eQTLs produced in this work with those identified in *Wang* et al. [40].

### Functional enrichment analysis

The tool GREAT [54] was used to perform enrichment analyses of biological pathways and networks as well as disease functions associated with genes present in regions nearby TEVs, regions nearby TEVs associated to a *cis*-eQTL and genomic regions enriched in TEVs from the sliding windows analysis. Set genes were uploaded on the GREAT web platform (version 3.0.0) in order to identify functionally enriched gene categories. A FDR threshold of 0.1 was used for this purpose.

### Intra-genic TEVs orientation analysis

Active TEs can be inserted inside coding genes in the same or opposite strand with respect to the transcribed strand. We attempted to assess whether different classes of polymorphic TEs are enriched or depleted for either kind of orientation. We defined as concordant TEVs those showing transcription in the same orientation of the gene they are inserted into, and as discordant those whose orientations are opposite. Upon selecting TEVs located into coding genes by choosing only elements which overlapped with Ensembl release 92 [55] genes using bedtools [51], we assessed the percentage of concordant and discordant TEVs separately for each one of the three classes of TE.

### TEVs enrichments in regulatory regions analysis

In order to assess whether different classes of TEVs are enriched, depleted or randomly distributed with respect to human regulatory sequences, we selected all TEVs and intersected them with human regulatory features obtained from Ensembl release 92 [55] using bedtools [51]. Then, for each of the 3 classes of TEVs, 100 randomization experiments were performed in order to assess whether enrichments and depletions were significant. Results featuring a Z score > or < 3 were considered significant.

### Transcribed TEVs analsysis

We also aimed at exploring the possible transcription of polymorphic TEs as fragments fused to annotated transcripts. For this purpose we downloaded RNA-seq fastq data from the 445 individuals belonging to individuals from the 1000 Genome Project [39] from the Geuvadis website [48]. Reads were aligned to the human reference genome (hg38) with BWA (mem module, standard parameters) [56]. Alignment files were analyzed with MELT [57] in order to find non-reference transposable element insertions in the RNA-seq mapping data. The TE genomic coordinates of the identified insertions were intersected with the 1000 Genome Project annotated polymorphic TEs coordinates [39] using bedtools [51]. Only non-reference insertions of TEs call of the same kind (Alu, L1 or SVA) present in both RNA-seq MELT analysis and 1000 Genome Project annotated TEVs separated by less than 50 bp and identified in the same individual were considered as potentially expressed TEVs. All positions and genes associated with variants found were obtained from MELT output annotations.

## Results and discussion

### Identification of TEVs and their functional enrichments

**Fig. 1:**
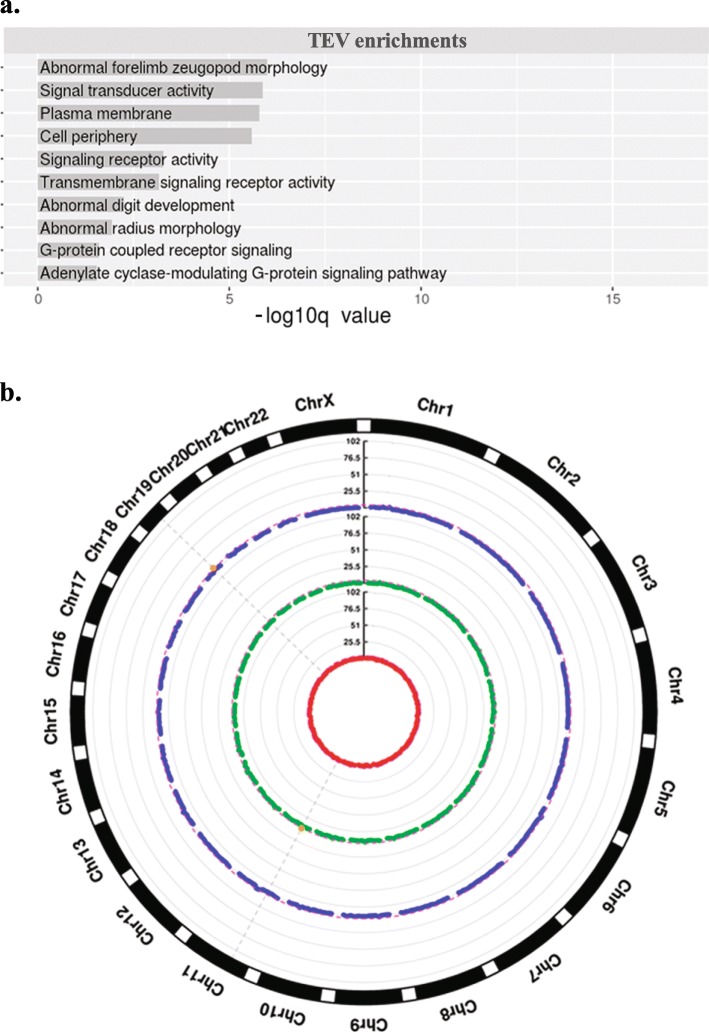
**a**. Functional enrichments related to all genes associated with TEVs against the whole genome. Enrichments are shown along with the FDR indicating the significance of the enrichments; **b**. Circular Manhattan plot representing genomic regions enriched in TEVs. Coordinates on the x-axis represent windows of 10 Mbp overlapping by 5 Mbp. On the y-axis the significance is reported as the negative log10 of the *p*-value from the randomization analysis for Alus (red), SVAs (blue), L1 s (green). Regions putatively enriched in TEVs (chr11:35000000–45,000,000 and chr19:35000000–45,000,000) are represented by orange dots, above the significance line (FDR = 0.05)

The final release of the 1000 Genome Project uncovered the location of thousands of variants resulting from TE activity (TEVs) in 25 populations [39]. We analyzed TEVs identified from whole genome sequencing of 445 healthy individuals for which RNA-seq was performed as well [48]. In this set, there are a total of 11,579 TEVs, comprising 9605 Alus, 1430 L1 s and 544 SVAs. In order to extend previous analyses, we decided to take into account all TEVs present in at least 3 out of 445 individuals, which resulted in a MAF of 0.7%. Although this choice might increase the number of potential false positives, it would give a broader vision, which is more consistent with our aim of performing an exploratory analysis. We also included those SVs which are labeled as *deletions* in the 1000 Genome Project data and are named as ALU_DEL, LINE1_DEL or SVA_DEL in the *info* column of the 1000 Genome Project VCF file. They correspond to TEVs polymorphisms where the TE is absent in the reference genome and therefore classified as deletion. With the inclusion of the 1291 *deletions* and the exclusion TEVs present in less than 3 individuals, the resulting subset of TEVs consisted of 8551 total TEVs (7208 Alu, 937 L1 and 406 SVA) (Table 1, Additional file 2). Functional enrichment analyses on the genes located in genomic regions containing these variants revealed significant functional enrichment, with respect to the whole genome, for several classes of receptors and signaling activity and genes involved in developmental abnormalities (Fig. 1a). This result is not based on expression data and therefore it is not biased by the cell-line used for the sequencing. It suggests a putative role for TE polymorphism in the generation of variability in morphological and developmental traits and is consistent with recent works proposing a contribution of TEs to regulatory innovations in mammalian embryonic and pluripotent states [58], which prevents their complete repression by the host genome.

**Table 1:** For each of the three TE classes we report the number of TE fragments fixed in the human reference genome (hg38), those in the set used for our eQTL analysis and those displaying significant *cis*-eQTL associations. ES: extended set

	Reference	TEV ES	TEV ES *cis*-eQTL
Alu	1,186,920	7208	267
L1	1,001,258	935	21
SVA	5750	406	35
Total	2,193,928	8551	323

### TEVs are mostly homogeneously distributed in the human genome

We then assessed whether specific genomic regions are enriched in TEVs in comparison to a random model and therefore divided the human genome in sliding windows of 10 Mbp overlapping for 5 Mbp looking for segments enriched for either one of the three classes of TEs. We used a *sliding windows* approach in order to reduce biases and sampling noise in comparison to analysis approaches based on individual locations [52]. We found only two genomic regions characterized by a slight significant enrichment for TEVs: chr19:35000000–45,000,000 for SVA and chr11:35000000–45,000,000 for L1 (Fig. 1b). We also performed functional enrichment analyses with GREAT [54] on the genes present in these regions featuring TEVs enrichment. However, no functional enrichment has been found in comparison with the whole genome. We conclude that, overall, TEVs are homogeneously distributed in the human genome.

### Identification of TEV-related expression quantitative trait loci in *cis* (*cis*-TEV-eQTL)

We produced associations between polymorphic TEs and gene expression in *cis*, searching for genes showing a significant alteration in their expression levels in individuals carrying the polymorphisms compared to individuals not carrying it, in a range of 1 Mbp from each TEV. This analysis allowed us to identify 323 significant TEVs *cis*-eQTL associations (FDR < = 0.05). This result is also significantly higher than the number of the average significant results from 100 randomizations (average randomization results = 76.5, Z > 50, *p* < 1e-170). A complete list of the *cis*-eQTL is available in Additional file 3. In a previous work on the same population, *Wang* et al. identified 83 *cis*-eQTLs analyzing TEVs featuring MAF > 5% (2617 total TEVs) [48]. We will therefore refer to our TEV dataset as Extended Set (ES). The higher number of eQTLs we found is due to the higher number of variants analyzed (8551) and to the choice to take into account also variants with an allelic frequency < 5% (variants common to at least 3 individuals out of 445). In our results, we found a total of 83 *cis*-eQTLs with MAF > = 5%, all of which were in overlap with the 83 identified by *Wang* et al. (Additional file 4). In addition, the proportion of significant *cis*-eQTL was comparable between the two studies (3% vs 3.7%, *p*-value = 0.17) suggesting that the choice of a different MAF did not significantly increase the number of false positives in our analysis.

**Fig. 2:**
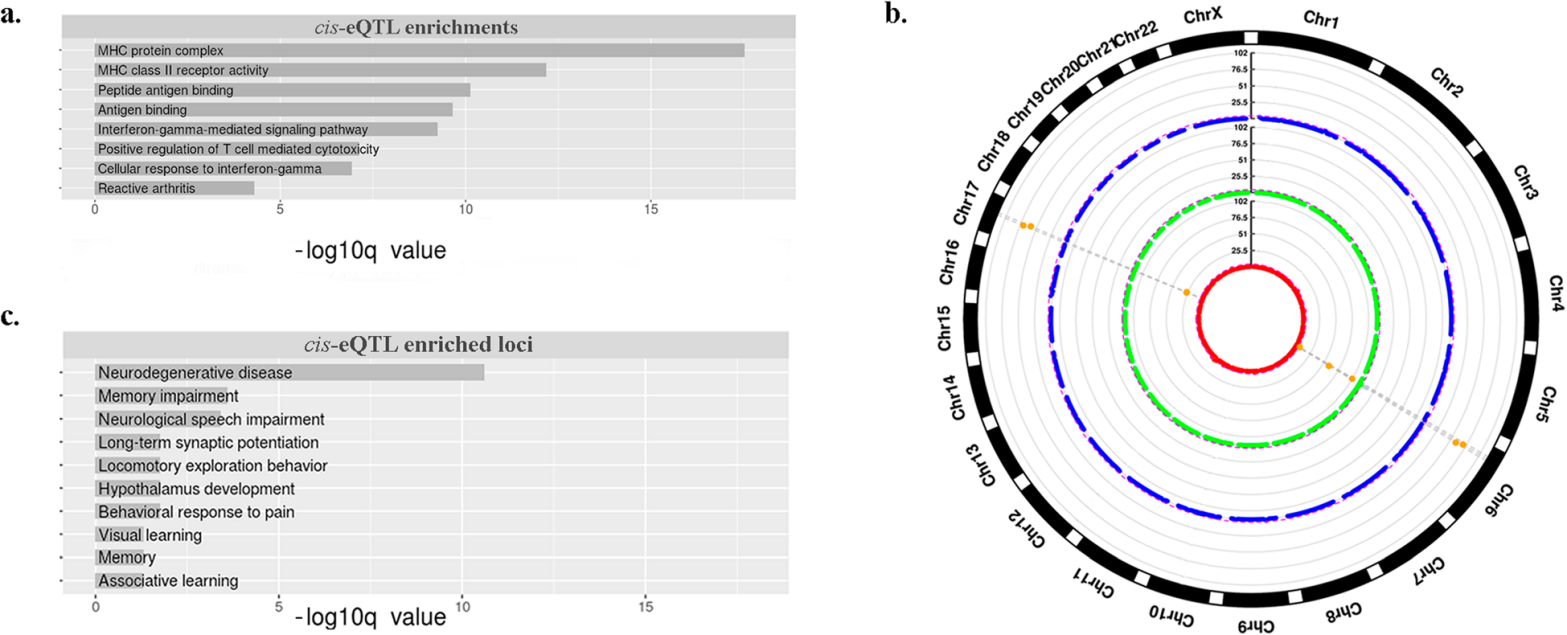
**a**. Functional enrichments related to all genes associated with TEV-*cis*-eQTL against all TEVs, enrichments are shown along with the FDR indicating the significance of the enrichments related to all genes associated with TEV-cis-eQTL against all TEVs; **b**. Circular Manhattan plot representing genomic regions enriched in TEVs-*cis*-eQTLs. Coordinates on the x- axis represent windows of 10 Mbp overlapping by 5 Mbp, while the negative log10 of the p-value associated to the TEV-*cis*-eQTL enrichments within each 10 Mbp window is plotted on the y-axis for Alus (red), SVAs (blue), L1 s (green). Regions putatively enriched are represented by orange dots, above the significance line (FDR = 0.05); **c.** Functional enrichments related to genes found in the two loci enriched in TEV-*cis*-eQTL (6p21.32 and 17q21.31). Enrichments are shown along with the FDR indicating the significance value

We performed functional enrichment analyses of genes related to the identified *cis*-eQTLs with respect to genes nearby all TEVs with GREAT [54]. The vast majority of significant enrichments (FDR < = 0.1) are related to the immune system and chromatin remodeling (Fig. 2a). This result is in line with the fact that the expression data are relative to LCL cell lines, and therefore possibly suggests a cell-type-specific functional relevance of the identified TEVs. We also performed a similar enrichment analysis for each distinct class of TEVs against the whole TEV-eQTLs set without finding any significance. This may be due to the small sample size of individual classes of TEV-*cis*-eQTL.

### The identified TEV-*cis*-eQTL are enriched in two specific genomic loci associated to immunity and behavior

Although TEV-gene associations are spread throughout the genome, we wanted to know whether some regions were enriched in the identified TEV-*cis*-eQTL. To this aim, we divided the genome into 10 Mbp windows overlapping by 5 Mbp, and assessed whether some of these regions result enriched in *cis*-eQTL in comparison to a random model. Results indicate the existence of two *hot-spots* showing a significantly higher frequency of *cis*-eQTLs: 6q21.32 and 17q21.31 (Fig. 2b). These regions appear strongly enriched for Alu and SVA related *cis*-eQTLs (Z > 50, *p* < 1e-60). Genes mapping into these loci, are mostly related to the immune response and the nervous system. Locus 6q21 contains most of the genes encoding for the human leukocyte antigene (HLA), cell-surface proteins responsible for the regulation of the adaptive immune system [59]. Such genes are themselves very polymorphic [60] and the different alleles allow for a fine-tuning of the immunological response. Locus 17q21.31 contains several genes potentially implicated in neurodegenerative diseases [61] and strongly tied to other dementias [62, 63]. Due to its structure, it is prone to genomic rearrangements [64, 65] whose evolutionary meaning is not yet clear. Interestingly, the importance of this locus for cognition potentially increased along mammalian evolution. In fact, while in mouse some of these genes (i.e. the LRRC37 gene family) show a testis-specific expression, in primates they also present a pattern of expression in regions of the brain [66]. Moreover, copy number variants (CNVs) in 17q21.31, have been shown to affect neuronal differentiation of hPSC cellular phenotype [67], likely through the WNT3/WNT9B pathway. This region contains also the gene KANSL1, which encodes for a chromatin modification protein with histone acetyltransferase activity and known to be strongly associated to multiple types of dementias. For example, all the phenotypic manifestations related to the 17q21.31 microdeletion syndrome can be caused by either a 400–800 kb long deletion in the 17q21.31 locus or by point mutations in KANSL1 alone [68–70]. In a recent work, mouse models of 17q21.31 microdeletion, microduplication syndromes and KANSL1 KO have been used to highlight the importance of KANSL1 for social behaviors, object recognition, and fear conditioning memory [71]. CNVs in locus 17q21.31 have also been associated to dyslexia [72], this is believed to be due to the variations affecting the gene NSF [72]. Taken together, these findings support an involvement for 17q21.31 genes in complex behavior, suggesting that variation in such locus could be linked to behavioral and cognitive differences among individuals. This possible association is strengthened by GWAS associations concerning food and drug addiction [73], smoking habits [74] and alcohol consumption [75] which have been found enriched within locus 17q21.31. Altered expression levels of two members of the LRRC37 gene family (LRRC37A and LRRC37A2), as well as KANSL1 and its antisense transcript KANSL1-AS1 are related to the presence of specific TEVs in our *cis*-eQTLs list. These genes comprise about half (18 on 35) of the *cis*-eQTLs found in locus 17q21.31 (Additional file 3).

We then performed functional enrichment analyses on genes involved in eQTL present in these two loci against the whole set of genes involved in TEV-*cis*-eQTLs. This analysis confirmed the enrichment in molecular functions and biological processes related to the immune response (such as MHC class II receptor activity and peptide antigen binding) and to neuronal activity, memory, behavior and neurological and neurodegenerative diseases (Fig. 2c). Considering that these enrichments are only due to primate-specific TEs (Alu and SVA), these results suggest a potential involvement of the activity of these TE classes in the diversification of primate- and human-related immune and CNS features. The association between the immune and the nervous systems has already been proposed at the genic level by the observation that increased brain size in mammals is also associated with size variation in gene families involved in the immune system [76]. We therefore speculate that primate-specific TEVs, could have contributed to both the recent evolution and phenotypic differences among individuals of immunity-related and behavioral traits.

**Fig. 3:**
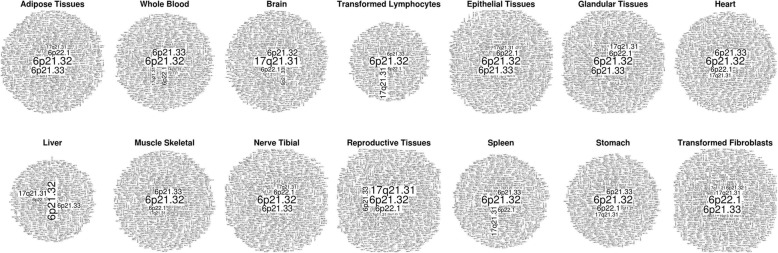
Word cloud representing the human loci featuring the highest density of GTEx SNP-*cis*-eQTLs in multiple tissues, data normalized for the size of each karyotype band

We decided also to assess whether specific chromosome bands result enriched for tissue-specific eQTL. To this aim, we downloaded the SNP-based eQTL data from the GTEx project (V7), which comprise associations from 44 human tissues belonging to 449 donors. We then counted the number of SNP-based *cis*-eQTLs found in each karyotype band for each tissue. Strikingly, while for almost all tissues, the loci on chromosome 6 were the top enriched in eQTL, the locus 17q21.31 was the top enriched in eQTLs specifically for the brain (Fig. 3). This gives support to the idea that variations in 17q21.31 are probably linked to cognitive phenotypes.

### Relative contribution of polymorphic SVAs to TEV-*cis*-eQTLs is significantly higher with respect to L1 s and Alus

**Fig. 4:**
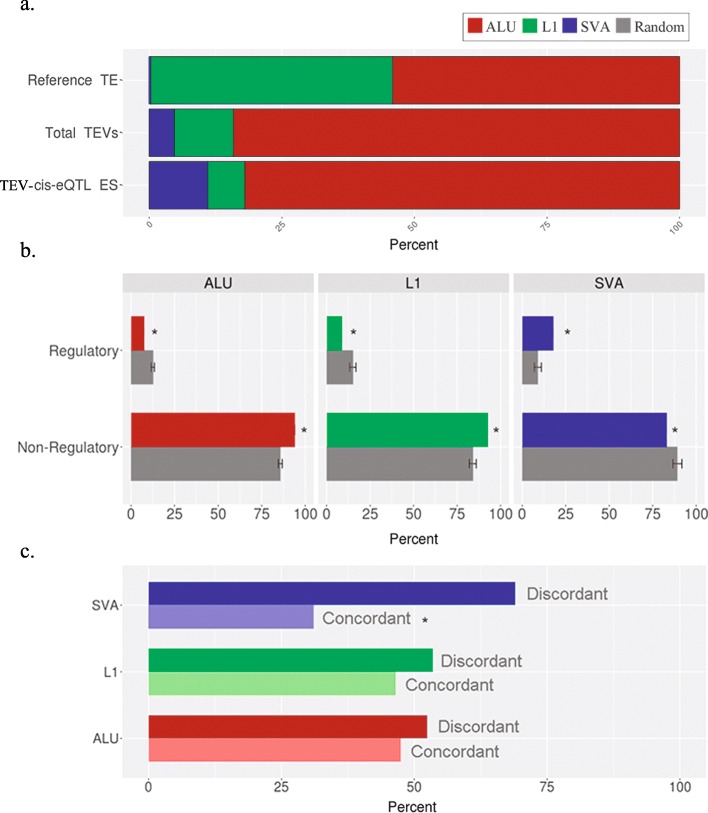
**a**. Percentage distribution of the three classes of TE in the reference genome, among all TEVs and among TEV-*cis*-eQTLs; **b**. percentage of TEVs overlapping regulatory and non-regulatory regions against random distributions (*Z-score < − 3, > 3); **c.** percentage of intra-genic TEVs on the same and opposite strand with respect to the transcribed strand for each class of TEVs (Fisher test, *p* < 0.00001)

We then explored the relative contributions of the three different element classes (L1, Alu and SVA) to the identified *cis*-eQTLs to understand whether a specific class is more frequently associated to changes in gene expression. Overall, Alu-derived TEVs provide the greatest number of *cis*-eQTL by far, consistently with their substantially higher number of copies in the human genome. However, the proportion of class-specific TEs resulting in TEV-*cis*-eQTLs is significantly higher for SVA compared to both Alu and L1 variants (Fisher test, *p* < 0.0001) (Fig. 4a). The same enrichment is found when performing the same analysis on *cis*-eQTLs extracted from *Wang* et al. [40]. This result suggests a stronger effect of SVA in generating phenotypic variation in humans with respect to what was previously thought. On the other hand, both the proportion of Alus and L1 s associated to eQTLs are either not significantly enriched or even depleted with respect to the entire set of TEV-ES.

### Polymorphic SVAs are enriched in regulatory regions

In order to evaluate the regulatory potential of the identified TEV-*cis*-eQTLs, we assessed whether the three classes of TEVs display any preferential genomic location with respect to annotated regulatory sequences. To this purpose, we downloaded the coordinates of human regulatory features from Ensembl [55] and intersected all three TEV classes with them. For each class we performed 100 randomizations in order to compute a Z score statistic. Results featuring a Z score < − 3 or > 3 were considered respectively significantly enriched or depleted. Interestingly, SVAs where enriched in regulatory sequences, and depleted in non-regulatory sequences compared to the randomizations, while the opposite was obtained for Alus and L1 s (Fig. 4b). These results could explain the relative higher contribution of SVA to *cis*-eQTLs. By analyzing many SV classes as a single group, previous works suggested a depletion of polymorphic SVs in regulatory regions [39]. Our results are novel and suggest that specific classes of polymorphic SVs might instead have an important regulatory impact.

### Concordant polymorphic intra-genic SVAs are depleted compared to discordant ones

We then investigated the orientation of intra-genic TEVs in regards to the strand of transcription of the gene they were inserted into. In Fig. 4c we plotted the percentage of TEVs found in the same (concordant) or opposite (discordant) strand with respect to the strand of transcription of the host gene. Interestingly, we found that the proportion of polymorphic intra-genic SVA insertions on the discordant strand is much higher compared to polymorphic L1 s and Alus (Fig. 4c). This result suggests the existence of a stronger selection against insertions on the same strand of transcription for polymorphic SVA insertions. We hypothesize that this might be due to a stronger regulatory influence of concordant SVAs on the host genes. Given the younger age of SVAs, it is reasonable to speculate that the human genome has not developed yet an efficient molecular mechanism capable to silence the effects of SVA insertions, conversely to what has happened for L1 s. These observations are consistent with the observation that SVAs are evolutionarily the most recent class of TEs [32] and could therefore be a major generator of inter-individual phenotypical differences.

### Evidences of transcription for some TEVs

**Fig. 5:**
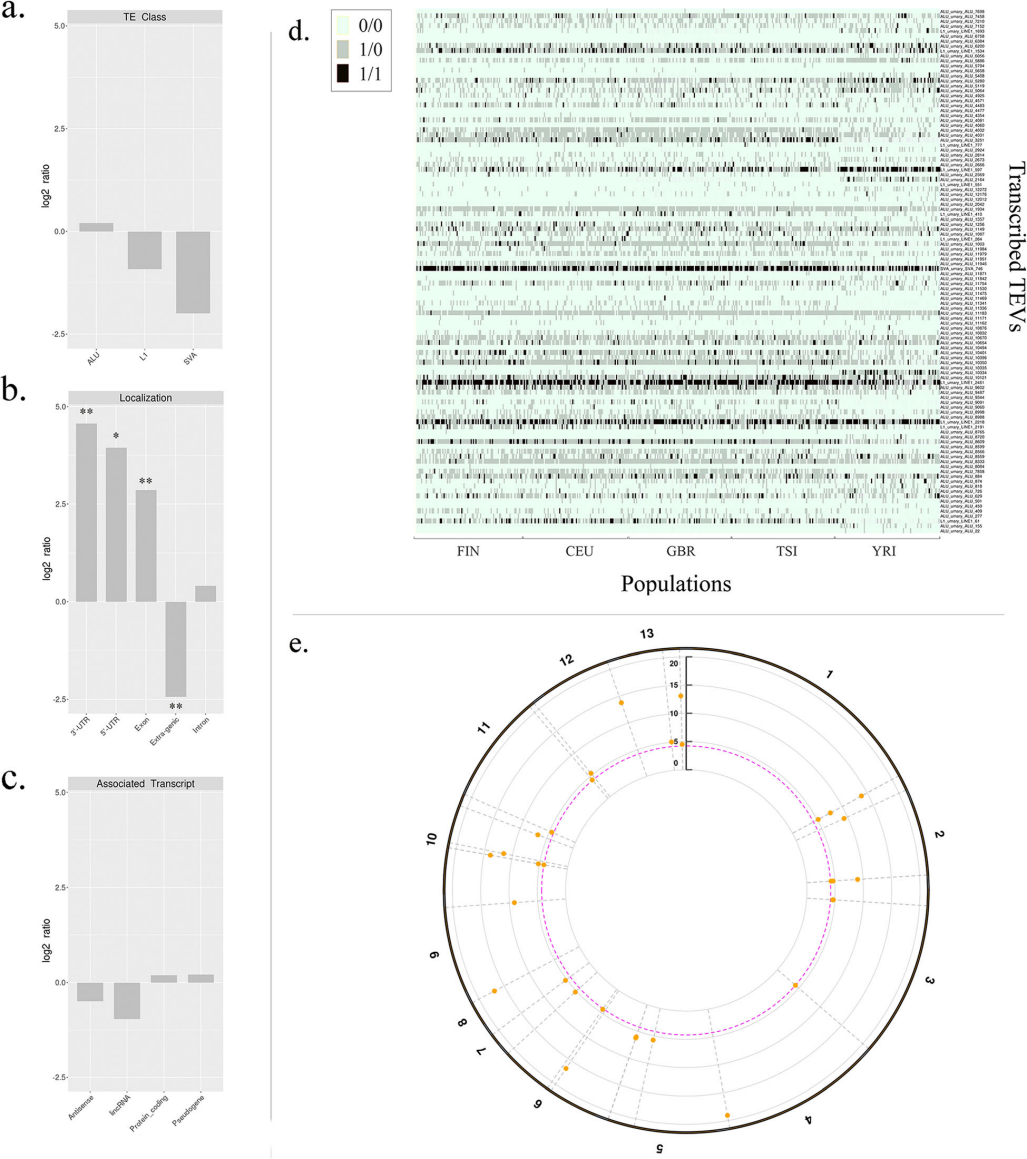
Putatively transcribed TEVs: **a**. Log2 of the ratio between the percentage of RNA-TEV and total TEV for each TEV class; **b**. Log2 of the ratio between the percentage of RNA-TEV and total TEV embedded in different genomic locations (Fisher test, ** = p-value < 0.0001, * = p-value < 0.001); **c**. Log2 of the ratio between the percentage of RNA-TEV associated to different gene types and the percentage composition of the total transcriptome for each transcript type; **d**. heatmap representing the distribution of RNA-TEVs among the individuals of the 5 analysed populations, the population are displayed on the x-axis, while the RNA-TEV are plotted on the y-axis **e**. Circular Manhattan plot representing the RNA-TEV associated to an altered gene expression, genomic coordinates are plotted on the x-axis while the negative log10 of the p-value associated to each RNA-TEV-gene association is displayed on the y-axes, significant associations are represented as orange dots above the significance line (FDR = 0.05)

We then assessed whether TEVs could be transcribed as part of annotated coding transcriptional units. We therefore run a MELT [57] analysis using RNA-seq data against the reference human genome of the same 445 individuals from whose we produced *cis*-eQTLs. We found 113 total TEVs present in both DNA and RNA sequencing data from the same individuals (RNA-TEVs). Most of them are Alus (100 out of 113) (Additional file 5), with 12 L1 s and 1 SVA. The proportion of Alus in this dataset is higher than the one in the whole TEVs set (Fig. 5a). Evolutionary fixed Alus insertions are known for their involvement as donor of splice sites [18] and signals for RNA-editing [19]. In addition, their pervasive and preferential exonization, with respect to other TE families, is also known [77]. Here we show the first evidence of such process relatively to polymorphic Alus.

The genomic localization of the identified potentially exonized polymorphic elements varies, with intronic being the most frequent localization (~ 60%), followed by 3′-UTR (~ 30%). Considering the enrichments with respect to the localization of the complete set of TEVs, the 3′-UTR stands out as being the most significantly enriched (Fig. 5b) suggesting that integrations and potential *gain-of-functions* are better tolerated in this region and might more easily constitute non-deleterious variations for the host. 5′-UTR and exons are also significantly enriched. These results are consistent with previous works on evolutionarily fixed TE elements, describing the involvement of TE, principally Alus, in exonization [77], miRNA interaction [18, 78], RNA editing [19, 20] and translational regulation [21, 22]. Most of RNA-TEVs are associated to coding genes (98 on 113, ~ 85%) and are embedded inside their associated gene. The proportion of RNA-TEVs associated to protein-coding genes and pseudogenes is also higher with respect to antisense and lincRNA transcripts which are less represented in RNA-TEVs compared to all TEVs (Fig. 5c). About half of these potentially transcribed TEVs features an allelic frequency > 5% in the analyzed population, suggesting positive selection of these elements. To further support this hypothesis, we plotted the presence/absence of each RNA-TEV (Fig. 5d). The resulting heatmap confirms the grouping of some RNA-TEVs among European or African individuals. Furthermore, some of the putatively transcribed polymorphic TEs are associated with a significantly altered expression of the overlapping gene (33 out of 113) in lymphoblastoid cell lines being also part of the TEV-*cis*-eQTL results (Fig. 5e). Therefore some RNA-TEVs might also be associated to changes in transcript levels.

## Conclusions

The impact of TEs, both polymorphic and fixed in the genome, has recently started to be analyzed to some extent [12, 40], however, the specific contribution to gene regulation, structural distribution, regional enrichments and potential transcription of different TE classes still need to be thoroughly explored. Here we present an analysis centered on the exploration of such features among the three classes of human active TEs. We also extended the analysis to less common and rare variants important for their potential involvement in uncharacterized genetic risk underlying diseases.

Our results indicate that two genomic regions, locus 6q21.32 and 17q21.31, are enriched in TEV-related *cis*-eQTL. These regions include a plethora of genes involved in immune response and cognitive functions. Considering that such enrichments are only due to the primate-specific TEs (Alu and SVA), it is reasonable to suggest that these evolutionarily young TEs played an important impact in the recent evolution of the immune system and cognition, promoting a degree of inter-individual differences. Importantly, SVAs present the highest relative impact on gene regulation. Furthermore, intra-genic polymorphic SVA insertions oriented discordantly with respect to the transcription of the host gene are significantly more frequent compared to intra-genic TEVs oriented concordantly. This divergence is conserved at a lower level for other classes of retrotransposons, suggesting a stronger selection against insertions on the strand of transcription for SVAs. Moreover, we began to uncover the existence and the impact of TEVs which might be transcribed as a part of known transcriptional units, mainly coding. We speculate that specific TEVs could promote the formation of individual-specific transcripts, potentially contributing to unique phenotypic traits.

Overall, our results contribute to increase the knowledge on the impact of TEs on gene expression in human. Since our analyses have been limited to one cell-line, it would be of great interest to perform similar analysis on multiple tissues and cell lines provided by public databases and consortia such as those from the GTEx project [44]. Finally, while our results are based on association studies, we have provided a list of candidates TEVs for experimental validation to establish a causal relationship between TEVs, transcriptional changes and variable human phenotypes. This work will be important in further defining the role of TEs in human evolution, diversification and their contribution to disease.

## Supplementary information

**Additional file 1:** Table containing the tissue grouping used for assessing the loci most enriched in SNP-eQTLs for each tissue group.

**Additional file 2: **Table containing all TEVs used for the *cis*-eQTL analysis.

**Additional file 3: **Table containing the significant TEV-*cis*-eQTLs.

**Additional file 4: **Venn diagram showing the intersection between *cis*-eQTL found in this work and in *Wang* et al.

**Additional file 5:.** Table containing the putatively transcribed TEs as part of annotated transcripts.

## Data Availability

All analyses have been performed on publicly available data. Namely: 1000 Genome Project phase III (ftp://ftp.1000genomes.ebi.ac.uk/vol1/ftp/phase3/integrated_sv_map/), GTEx version 7 (https://gtexportal.org/home/datasets) and GEUVADIS consortia (ftp://ftp.ebi.ac.uk/pub/databases/microarray/data/experiment/GEUV/E-GEUV-1/analysis_results).

## Abbreviations

CNS: Central nervous system
eQTL: Expression quantitative trait loci
FDR: False discovery rate
LCL: Lymphoblastoid cell line
MAF: Minor allele frequency
Mbp: Mega base pairs
SNP: Single nucleotite polymorphism
SV: Structural variants
SVA: SINE-R/VNTR/Alus
TE: Transposable elements
TEV: Transposable elements variants

## References

[1] McClintock B (1950). The origin and behavior of mutable loci in maize. Proc Natl Acad Sci.

[2] Mills RE, Bennett EA, Iskow RC, Devine SE (2007). Which transposable elements are active in the human genome?. Trends Genet.

[3] Notwell JH, Chung T, Heavner W, Bejerano G (2015). A family of transposable elements co-opted into developmental enhancers in the mouse neocortex. Nat Commun.

[4] Slotkin RK, Martienssen R (2007). Transposable elements and the epigenetic regulation of the genome. Nat Rev Genet.

[5] Stavenhagen JB, Robins DM (1988). An ancient provirus has imposed androgen regulation on the adjacent mouse sex-limited protein gene. Cell.

[6] Mariño-Ramírez L, Lewis KC, Landsman D, Jordan IK (2007). Transposable elements donate lineage-specific regulatory sequences to host genomes. Cytogenet Genome Res.

[7] Medstrand P, Van De Lagemaat LN, Dunn CA, Landry JR, Svenback D, Mager DL (2005). Impact of transposable elements on the evolution of mammalian gene regulation. Cytogenet Genome Res.

[8] Kim SY, Pritchard JK (2007). Adaptive evolution of conserved noncoding elements in mammals. PLoS Genet.

[9] Maka W (2000). Genomic scrap yard: how genomes utilize all that junk. Gene.

[10] Jordan IK, Rogozin IB, Glazko GV, Koonin EV (2003). Origin of a substantial fraction of human regulatory sequences from transposable elements. Trends Genet.

[11] Conley AB, Piriyapongsa J, Jordan IK (2008). Retroviral promoters in the human genome. Bioinformatics.

[12] Trizzino M, Park Y, Holsbach-beltrame M, Aracena K (2017). Transposable elements are the primary source of novelty in primate gene regulation. Renome Res.

[13] Kunarso G (2010). Transposable elements have rewired the core regulatory network of human embryonic stem cells. Nat Genet.

[14] S. Francisco, S. Cruz, and C. T. View, “A distal enhancer and an ultraconserved exon are derived from a novel retroposon,” Nature, no. January 2014, 2006;441:87-90. 10.1038/nature04696 16625209

[15] Conley AB, Jordan IK (2012). Cell type-specific termination of transcription by transposable element sequences. Mob DNA.

[16] Gal-mark N, Schwartz S, Ast G (2008). Alternative splicing of Alu exons - two arms are better than one. Nucleic Acids Res.

[17] Sorek R, Ast G, Graur D (2002). Alu -containing exons are alternatively spliced. Genome Res.

[18] Daskalova E, Baev V, Rusinov V, Minkov I (2006). 3’UTR-located ALU elements: donors of potential miRNA target sites and mediators of network miRNA-based regulatory interactions. Evol Bioinformatics Online.

[19] Daniel C, Silberberg G, Behm M, Öhman M (2014). Alu elements shape the primate transcriptome by cis-regulation of RNA editing. Genome Biol.

[20] Rosenthal JJC, Seeburg PH (2012). A-to-I RNA editing: effects on proteins key to neural excitability. Neuron.

[21] Carrieri C (2012). Long non-coding antisense RNA controls Uchl1 translation through an embedded SINEB2 repeat. Nature.

[22] Schein A, Zucchelli S, Kauppinen S, Gustincich S, Carninci P (2016). Identification of antisense long noncoding RNAs that function as SINEUPs in human cells. Sci Rep.

[23] Trizzino M, Kapusta A, Brown CD (2018). Transposable elements generate regulatory novelty in a tissue-specific fashion. BMC Genomics.

[24] Mager DL, Medstrand P (2003). Transposable elements in mammals promote regulatory variation and diversification of genes with specialized functions. Trends Genet.

[25] Ostertag EM, Kazazian HH (2001). Biology of mammalian L1 retrotransposons. Annu Rev Genet.

[26] Raiz J (2012). The non-autonomous retrotransposon SVA is trans-mobilized by the human LINE-1 protein machinery. Nucleic Acids Res.

[27] Esnault C, Maestre J, Heidmann T (2000). Human LINE retrotransposons generate processed pseudogenes. Nat Genet.

[28] Wicker T (2009). A unified classification system for eukaryotic transposable elements. Nat Rev Genet.

[29] Hormozdiari F., Alkan C., Ventura M., Hajirasouliha I., Malig M., Hach F., Yorukoglu D., Dao P., Bakhshi M., Sahinalp S. C., Eichler E. E. (2010). Alu repeat discovery and characterization within human genomes. Genome Research.

[30] Ran C (2010). Mobile interspersed repeats are major structural variants in the human genome. Cell.

[31] Witherspoon DJ (2009). Alu repeats increase local recombination rates. BMC Genomics.

[32] Ostertag EM, Goodier JL, Zhang Y, Kazazian HH (2003). Report SVA elements are nonautonomous retrotransposons that cause disease in humans. Am J Hum Genet.

[33] Ono M, Kawakami M, Takezawa T (1987). A novel human nonviral retroposon derived from an endogenous retrovirus. Nucleic Acids Res.

[34] Savage AL (2014). An evaluation of a SVA retrotransposon in the fus promoter as a transcriptional regulator and its association to ALS. PLoS One.

[35] O. Vasieva, S. Cetiner, A. Savage, G. G. Schumann, V. J. Bubb, and J. P. Quinn, “Primate specific retrotransposons, SVAs, in the evolution of networks that alter brain function,” p. 22, 2016.

[36] Taniguchi-Ikeda M (2011). Pathogenic exon-trapping by SVA retrotransposon and rescue in Fukuyama muscular dystrophy. Nature.

[37] Barreiro LB, Laval G, Quach H, Patin E, Quintana-Murci L (2008). Natural selection has driven population differentiation in modern humans. Nat Genet.

[38] 1000 Genomes Project Consortium (2015). A global reference for human genetic variation. Nature.

[39] Sudmant PH (2015). An integrated map of structural variation in 2,504 human genomes. Nature.

[40] Wang L, Rishishwar L, Mariño-Ramírez L, Jordan IK (2017). Human population-specific gene expression and transcriptional network modification with polymorphic transposable elements. Nucleic Acids Res.

[41] Altshuler DL (2010). A map of human genome variation from population-scale sequencing. Nature.

[42] Farrall M (2004). Quantitative genetic variation: a post-modern view. Hum Mol Genet.

[43] Rockman MV, Kruglyak L (2006). Genetics of global gene expression. Nat Rev Genet.

[44] Book T (2015). Human genomics. The genotype-tissue expression (GTEx) pilot analysis: multitissue gene regulation in humans. Science.

[45] Brem RB, Yvert G, Clinton R, Kruglyak L (2002). Genetic dissection of transcriptional regulation in budding yeast. Science (80- ).

[46] Tung J, Zhou X, Alberts SC, Stephens M, Gilad Y (2015). The genetic architecture of gene expression levels in wild baboons. Elife.

[47] Doss S, Schadt EE, Drake TA, Lusis AJ (2005). Cis-acting expression quantitative trait loci in mice. Genome Res.

[48] Lappalainen T (2013). Transcriptome and genome sequencing uncovers functional variation in humans. Nature.

[49] Gibson G, Powell JE, Marigorta UM (2015). Expression quantitative trait locus analysis for translational medicine. Genome Med.

[50] Lonsdale J (2013). The genotype-tissue expression (GTEx) project. Nat Genet.

[51] Quinlan AR, Hall IM (2010). BEDTools: a flexible suite of utilities for comparing genomic features. Bioinformatics.

[52] Beissinger TM, Rosa GJ, Kaeppler SM, Gianola D, De Leon N (2015). Defining window-boundaries for genomic analyses using smoothing spline techniques. Genet Sel Evol.

[53] Shabalin AA (2012). Matrix eQTL: ultra fast eQTL analysis via large matrix operations. Bioinformatics.

[54] McLean CY (2010). GREAT improves functional interpretation of cis-regulatory regions. Nat Biotechnol.

[55] Zerbino DR (2018). Ensembl 2018. Nucleic Acids Res.

[56] Li H, Durbin R (2009). Fast and accurate short read alignment with burrows-wheeler transform. Bioinformatics.

[57] E. J. Gardner *et al.*, “The Mobile Element Locator Tool (MELT): Population-scale mobile element discovery and biology,” Genome Res., no. 410, 2017. 10.1101/gr.218032.116 PMC5668948 28855259

[58] Gerdes P, Richardson SR, Mager DL, Faulkner GJ (2016). Transposable elements in the mammalian embryo: pioneers surviving through stealth and service. Genome Biol.

[59] Meyer D, Vitor VR, Bitarello BD, Débora DY, Nunes K (2018). A genomic perspective on HLA evolution. Immunogenetics.

[60] dos Santos Francisco R (2015). HLA supertype variation across populations: new insights into the role of natural selection in the evolution of HLA-A and HLA-B polymorphisms. Immunogenetics.

[61] Cervera-Carles L (2016). Copy number variation analysis of the 17q21.31 region and its role in neurodegenerative diseases. Am J Med Genet Part B Neuropsychiatr Genet.

[62] Egloff M (2014). 17q21.31 microdeletion: brain anomalies leading to prenatal diagnosis. Cytogenet Genome Res.

[63] Grisart B (2009). 17Q21.31 microduplication patients are characterised by Behavioural problems and poor social interaction. J Med Genet.

[64] Fledel-Alon A, Leffler EM, Guan Y, Stephens M, Coop G, Przeworski M (2011). Variation in human recombination rates and its genetic determinants. PLoS One.

[65] Alves JM, Lopes AM, Chikhi L, Amorim A (2012). On the structural plasticity of the human genome: chromosomal inversions revisited. Curr Genomics.

[66] Bekpen C, Tastekin I, Siswara P, Akdis CA, Eichler EE (2012). Primate segmental duplication creates novel promoters for the LRRC37 gene family within the 17q21.31 inversion polymorphism region. Genome Res.

[67] Lee C (2015). WNT9B amplification in hPSCs with respect to amplification in hPSCs with respect to neural differentiation. Cell Rep.

[68] Moreno-Igoa M (2015). KANSL1 gene disruption associated with the full clinical spectrum of 17q21.31 microdeletion syndrome. BMC Med Genet.

[69] Zollino M (2012). Mutations in KANSL1 cause the 17q21.31 microdeletion syndrome phenotype. Nat Genet.

[70] Koolen DA (2016). The Koolen-de Vries syndrome: a phenotypic comparison of patients with a 17q21.31 microdeletion versus a KANSL1 sequence variant. Eur J Hum Genet.

[71] Arbogast T (2017). Mouse models of 17q21.31 microdeletion and microduplication syndromes highlight the importance of Kansl1 for cognition. PLoS Genet.

[72] Veerappa AM, Saldanha M, Padakannaya P, Ramachandra NB (2014). Family based genome-wide copy number scan identifies complex rearrangements at 17q21.31 in dyslexics. Am J Med Genet Part B Neuropsychiatr Genet.

[73] Cornelis MC (2016). A genome-wide investigation of food addiction. Obesity.

[74] Argos M (2014). Genome-wide association study of smoking behaviors among Bangladeshi adults. J Med Genet.

[75] Nelson EC (2011). H2 haplotype at chromosome 17q21.31 protects against childhood. Addict.

[76] Castillo-Morales A, Monzón-Sandoval J, Urrutia AO, Gutiérrez H (1775). Increased brain size in mammals is associated with size variations in gene families with cell signalling, chemotaxis and immune-related functions. Proc R Soc B Biol Sci.

[77] Sela N, Mersch B, Gal-Mark N, Lev-Maor G, Hotz-Wagenblatt A, Ast G (2007). Comparative analysis of transposed element insertion within human and mouse genomes reveals Alu’s unique role in shaping the human transcriptome. Genome Biol.

[78] R. Pandey, A. Bhattacharya, V. Bhardwaj, V. Jha, A. K. Mandal, and M. Mukerji, “Alu-miRNA interactions modulate transcript isoform diversity in stress response and reveal signatures of positive selection,” Sci Rep*.*, vol. 6, no. September, pp. 1–18, 2016. 10.1038/srep32348 PMC5009348 27586304

