# Supplementary figures and images for: Impact of polymorphic transposable elements on transcription in lymphoblastoid cell lines from public data
Source: BMC Bioinformatics. 2019 Nov 22;20(Suppl 9):495. doi: 10.1186/s12859-019-3113-x (PMC6873650; doi:10.1186/s12859-019-3113-x)

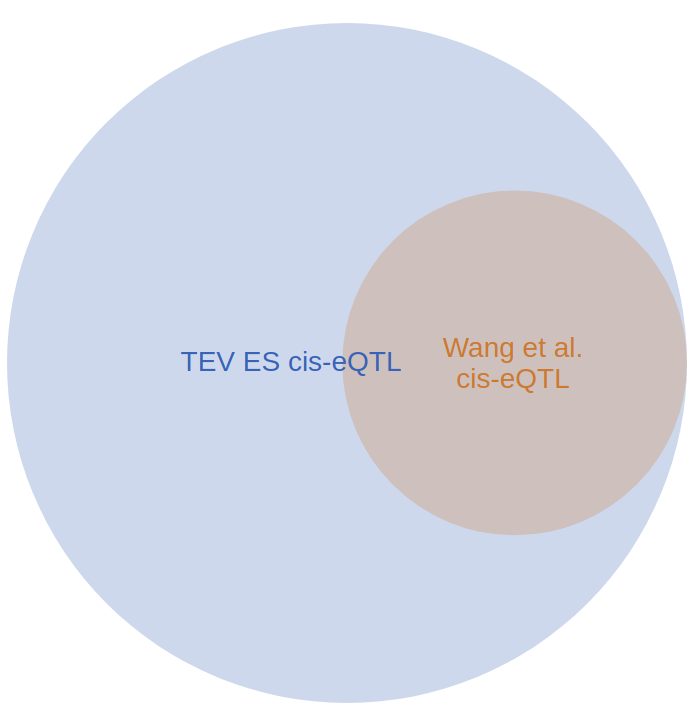

Supplement: Supplementary file 4 — Additional file 4: Venn diagram showing the intersection between cis-eQTL found in this work and in Wang et al. [file 12859_2019_3113_MOESM4_ESM.png]
